# A strategy for accurately and sensitively quantifying free and esterified fatty acids using liquid chromatography mass spectrometry

**DOI:** 10.3389/fnut.2022.977076

**Published:** 2022-08-03

**Authors:** Xiaohui Feng, Juan Wang, Zhonghai Tang, Bingyao Chen, Xinhua Hou, Jing Li, Shengnan Feng, Peng Li, Qingshi Meng

**Affiliations:** ^1^State Key Laboratory of Animal Nutrition, Institute of Animal Sciences, Chinese Academy of Agricultural Sciences, Beijing, China; ^2^Biotechnology Research Institute, Chinese Academy of Agricultural Sciences, Beijing, China; ^3^College of Food Science and Technology, Hunan Agricultural University, Changsha, China; ^4^China Animal Disease Control Center, Beijing, China

**Keywords:** fatty acids, isotopic-based derivatization, chemical derivatization, quantification, meat processing

## Abstract

Fatty acid (FA) composition of foods dictates a diversity of aspects regarding food quality, ranging from product shelf life, sensory properties to nutrition. There is a challenge to quantitate FAs using liquid chromatography-mass spectrometry due to poor ionization efficiency and matrix effects. Here, an isotopic-tagged derivatization strategy was established to accurately and sensitively quantify free and esterified FAs. After derivatization reaction, the detection sensitivity of FAs was remarkably improved and the limit of quantitation was lower than 100 ng/L. The quantitative errors caused by matrix effects were diminished benefiting from isotope-derivatized internal standards. The established quantitation strategy was successfully applied to verify both free and esterified FA contents in meat after different post-harvest procedures, finding that free polyunsaturated FAs increased significantly during freezing process.

## Introduction

Fatty acid (FA) profiles have closely associated with their nutrition and eating quality such as tenderness, shelf life, and flavor. FAs, which refers a group of compounds containing carboxyl group, can be categorized into three types of FAs on the basis of the number of double bonds, including saturated FAs (SFA), monounsaturated FAs (MUFA), and polyunsaturated FAs (PUFA) (1). Most of FAs are covalent bonded with alcohol and amine group forming fatty acyl, glycerollipids (GL), glycerophospholipids (GP), sphingolipid (SP), sterol lipid (ST), phenol lipids (PR), saccharolipids (SL), and polyketides (PK) (2). As major energy sources and membrane constituents, esterified FAs play important roles in regulating membrane structure and functions, intracellular signal pathway, gene expression and so on (3). Another type of FAs are free FAs, which can be either endogenously synthesized using protein and sugar or produced by lipolysis and oxidative reactions (4). The presence of free FAs has a direct influence on meat appearance, food quality and flavor. For example, SFA are associated with meat-fat firmness while MUFAs, cis-9-octadecenoic acid, are positive or linoleic acid is negative associated with meat appearance (5, 6). The sum of free FAs and esterified FAs from the circulating lipids, ranging from dietary intakes, breeding method, slaughter weight to meat post-harvest, may reflect the overall metabolism of endogenous and dietary FAs. Hence, it is essential to quantitative and qualitative analyze of both esterified and free FAs to satisfy the increasing demand on food safety and quality.

Gas Chromatography-Flame Ionization Detection (GC-FID) and Gas Chromatography-Mass Spectrometry (GC-MS) are the most commonly used platforms to quantitate FAs after a carboxyl group is transferred to ester group (4). Methylation and trimethyl-sialylation are two classic esterification methods. A variety of FA methylation methods are utilized including acid-based (MeOH–HCl, MeOH–H_2_SO_4_, and MeOH–BF_3_) and base-based (MeOH–NaOH or MeOH–KOH) catalyzation reactions (7). It should be noted that the FAs content obtained from these methods is the sum of free and esterified FAs. Moreover, low-volatility long-chain FA esters and the thermal instability of unsaturated FAs make GC analysis more complicated (8). To overcome these issues, Liquid Chromatograph-Mass Spectrometry (LC-MS) with high sensitivity character becomes a powerful alternative tool in FAs analysis. Direct analysis using LC-MS is conducted in negative mode, nevertheless it suffers poor ionization efficiency caused by carboxyl groups (9). To enhance ionization efficiency, chemical derivatization method provides an efficient way by the introduction of easy ionizable heteroatoms (10). Easy ionizable heteroatoms such as nitrogen and sulfur are induced to FAs via derivatization reactions (11). Additionally, the cyclic moiety of derivatization reagent confers hydrophobicity to FAs, enlarging their quantitation analysis in a wider dynamic range. Previous studies have used 3-nitropheylhydrazine (3-NP) to derivatize FAs. The formed 3-NP-derivatized FAs can be separated on reversed phase column and the detection sensitivity was enhanced compared with non-derivatized FAs due to the benzene ring and nitro-group (12). Due to the electronegativity of nitro-group, the 3-NP derivatives are detected under negative ESI mode, although the signal responses are weaker than positive ESI mode on most mass spectrometry platforms. Besides, matrix effect caused by the interference from complicated biological compounds needs to be considered. This phenomenon can be diminished via the introduction of isotope internal standard (IS). However, the commercially available isotope ISs are limited and expensive. Alternatively, the isotopic-coded derivatization reagents provide a way for FAs quantitation and the produced FA derivatives are utilized as ISs for quantitative analysis of FAs.

In this study, we succeeded to use a novel isotopic-coded derivatization reagent, 5-(dimethylamino)-1-carbohydrazide-isoquinoline (DMAQ), to accurately and sensitively quantify both free and esterified FAs. This reagent was synthesized and reported in our previous work (13), which had been successful used to quantify fatty aldehydes. The derivatization conditions regarding coupling reagents, additives, derivatization reagent, time, and temperature were optimized using FA standards and verified using meat extract. The optimized derivatization reaction was carried out under mild conditions and the derivatized FAs exhibited enhanced detection sensitivity and good separation performance. The established LC-MS quantitation method was validated via linearity, accuracy, precision and stability of the product. Matrix effects were also diminished using DMAQ-^13^C/^15^N-derivatized FAs as internal standards. For proof-of-concept study, the performance of the newly developed method was applied to detect free and esterified FA contents in meat after different post-harvest procedures.

## Materials and methods

### Chemicals and reagents

All FA standards [listed in Supplementary Table 1 in Supplemental Information (SI)] were purchased from Sigma-Aldrich (St. Louis, Mo, United States). Analytical-grade *N*-(3-Dimethylaminopropyl)-*N*’-ethylcarbodiimide hydrochloride (EDC), *N*, *N*’-Dicyclohexylcarbodi-imide (DCC), *N*, *N*′-Diisopropylcarbodiimide (DIC), 1-hydroxy-7-azabenzotriazole (HOAt), 1-hydroxylbenzotriazole monohydrate (HOBt), sodium hydroxide (NaOH), *N*, *N*-dimethylmethanamide (DMF), and other reagents were obtained from Aladdin Industrial Co., Ltd (Shanghai, China). HPLC-grade acetonitrile (ACN), isopropanol, methanol (MeOH), and hexane were supplied by Thermo Fisher Scientific (Rockford, United States). Both 5-(dimethylamino)-1-carbohydrazide-isoquinoline (DMAQ-^12^C/^14^N) and 5-[di(methyl-^13^C)amino-^15^N]isoquinoline-1-carbohydrazide (DMAQ-^13^C/^15^N) derivatization reagents were synthesized in-house (Supplementary Figure 1) (13).

### Derivatization optimization

Standards stock solution of FAs were prepared in ACN or hexane to obtain concentrations of 1 mg/mL. The mixed stock solution containing forty FAs standards were obtained by mixing an aliquot of FA standards in acetonitrile to provide final concentrations of 2 μg/mL. Ten-level mixed standards working solutions were produced through serial dilution of the resultant solution to provide concentrations of 0.1, 0.5, 1, 5, 10, 25, 50, 100, 250, 500, and 1,000 μg/L for method validation. The derivatization reagent, DMAQ-^12^C/^14^N or DMAQ-^13^C/^15^N, were dissolved in ACN to 20 mM. The coupling reagents included EDC, DCC, or DIC. EDC was dissolved in deionized water while DCC or DIC were diluted in DMF to 750 mM. The additive reagents, HOAt or HOBt, were dissolved in DMF to 15 mM. The derivatization solution includes the derivatization reagent, coupling reagent, additive reagent and DMF/ACN (4:1, ν:ν).

Briefly, 10 μL of the mixed standards solution was mixed with 10 μL of derivatization solution, followed by vortex mixing for 2 min. The mixture was incubated at 20°C for 1 h and 10 μL of 10 wt% formic acid was added to quench the derivatization reaction. The reaction mixture was concentrated under vacuo. After that, the sample was re-dissolved to 1 mL within ACN, which 2 μL of supernatant was injected into LC-MS. Using the above-mentioned approach, we firstly studied the influences of coupling-reagent types (EDC, DCC, and DIC) and their concentrations (250, 500, 750, and 1,000 mM), additive types (HOAt and HOBt) and their contents (15, 30, 45, and 60 mM), and DMAQ contents (4, 10, 20, and 40 mM) on the derivatization efficiency. Other reaction parameters regarding reaction temperatures (4, 20, 37, and 60°C) and times (15, 30, 60, and 90 min) were accessed. To determine whether the optimal reaction conditions were applicable to the complicated biological samples, meat was chosen as a biological model. Following the similar optimization procedure, we validated the derivatization reaction conditions on the basis of meat extract using DMAQ-^12^C/^14^N. The peak areas were employed to compare the conversion efficiency.

### Meat samples collection and processing

A total of five pig longissimus thoracis (LT) samples, about one-hundred gram for each sample, were bought and collected from slaughterhouse at Beijing Heiliu Stockbreeding Technology Co., Ltd., following close to the lines of “Operating procedures of livestock and poultry slaughtering-Pig (GB/T 17236-2019)” and OIE TAHC, CHAPTER 7.5. The pigs were slaughtered after stopping eat (free access to water) for 18 h, showering, electrical stunning, killing, dehairing, peeling, and sample collection. After collection, each LT sample was equally divided into four blocks and subjected to four different post-harvest processes. One block was immediately used to extract FAs, named as fresh meat. Two blocks were maintained at 4°C. After 24 h of incubation, one of them was used to extract FAs, named as chilled meat while the other chilled meat was stored at −20°C for another 1 week, named as chilled frozen meat. The rest block of fresh meat was stored under −20°C for 1 week, named as frozen meat. After different post-harvest processes, about ten grams of each sample was thawed, trimmed for adipose and connective tissue, and homogenized individually in meat grinder.

### Free and total fatty acid extraction and derivatization

Following the optimal derivatization procedure, the mixed FA standards solution were derivatized by DMAQ-^13^C/^15^N as internal standards (IS) and the obtained DMAQ-^13^C/^15^N-FAs solution was diluted to proper concentration according to the FAs contents in meat. Approximate 10.0 g of meat was minced using a meat grinder.

For free FAs extraction, about 1.0 g of meat sample were weighed and 4.9 mL of ice-cold acetonitrile and 0.1 mL of the IS solution were subsequently added. The mixture was homogenized using a tissue homogenizer. The obtained suspension was centrifuged at 14,000 rpm at 4°C for 10 min. The supernatant was collected and stored at −80°C before derivatization.

For total FAs extraction, 0.5 g of meat sample and 10.0 mL of 80% MeOH containing 0.5 M NaOH were added into a glass vial. The vial was capped with a silicon disk in a closed crew cap and incubated at 80°C for 3 h. After being cooled to room temperature, 1M HCl was added to adjust the reaction mixture to neutral condition, which was diluted within MeOH to 250 mL.

The above extracted FAs were derivatized by DMAQ-^12^C/^14^N following the optimal derivatization procedure. In brief, 50 μL of the extracted FAs from meat was mixed with 50 μL of derivatization solution containing 20 mM of DMAQ-^12^C/^14^N, 750 mM of EDC and 15 mM of HOAt. Then the reaction mixture was maintained at 20°C for 30 min. Finally, 10 μL of 10 wt% formic acid was added to quench the reaction and the reaction mixture was concentrated under vacuo. The obtained sample was redissolved in ACN before injecting into LC-MS.

### Liquid chromatograph-mass spectrometry analysis

Liquid chromatograph-mass spectrometry/mass spectrometry analysis of DMAQ-derivatized samples were carried out using an ACQUITY Ultra-Performance Liquid Chromatography (Waters, United States) coupled with a triple-quadrupole mass spectrometer (QTRAP 6500, United States). Two microliters of the derivatized sample were injected into LC-MS/MS system using an autosampler maintained at 4°C. An Agilent Eclipse Plus C8 RRHD 1.8 μm (2.1 × 100 mm, pore size 95 Å) at 45°C was used to separate target analytes in a sample. Mobile phase A was 0.1% (ν:ν) formic acid in water while mobile phase B was 0.1% formic acid (ν:ν) in isopropanol/acetonitrile (1:1, ν:ν). Elution was initiated with 70% A and 30% B. The 20-min gradient conditions were: 0–1.5 min (30–60% B), 1.5–11.0 min (60–75% B), 11.0–15.0 min (75–100% B), 15.0–18.0 min (100% B), 18.0–18.1 min (100–30% B), and 18.1–20.0 min (30% B). Flow rate was set at 400 μL min^–1^. The MS parameters were set as follows: ion-spray voltage of 5,500 V, turbo source gun temperature of 500°C and curtain gas of 30 psi. Data acquisition was performed in positive MRM mode. The ion transitions, collision energy (CE) and de-clustering potential (DP) were listed in Supplementary Table 2.

The discovery of product ions was carried out using a TripleTOF^®^ 6600 mass spectrometer (AB SCIEX; Framingham, MA, United States). The LC and ionization source conditions were consistent with that mentioned above. The mass range of MS was *m/z* 100–1,000 while that of MS/MS was in the range of *m/z* 50–1,000.

### Method validation

The proposed method was validated in the terms of linearity, sensitivity, accuracy, precision, stability, matrix effects (MEs), and recovery. For calibration curves, ten-level mixed standards solutions containing forty DMAQ-^13^C/^15^N-FAs were prepared through serial dilution of the mixed standards stock solution using ACN and the DMAQ-^12^C/^14^N-derivatized meat extract, respectively. Calibration curves were established by employing linear regression lines with the least-square fit. To validate the correlation of signal responses, the DMAQ-^13^C/^15^N-FAs was added into DMAQ-^12^C/^14^N-FAs to make the mixture at the ratios of 100:1, 10:1, 1:1, 1:10, and 1:100. The resultant mixture was then injected to LC-MS. The limit of detection (LOD) and limit of quantitation (LOQ) of each FA were estimated at the concentrations when the ratios of signal to noise (S/N) were close to three and ten, respectively. They were also demonstrated in ACN or meat extract, respectively. The accuracy and precision were accessed using inter- and intra-day assays. Following the free FAs extraction procedure mentioned in section “Free and total fatty acid extraction and derivatization,” FAs were extracted from meat using ACN containing proper concentration of DMAQ-^13^C/^15^N-FAs standards. The obtained extraction solution was derivatized in triplicate with DMAQ-^12^C/^14^N-derivatization reagent in a day and three consecutive days, respectively. The precision values were estimated using relative standard deviation (RSD). For stability study, six DMAQ-^12^C/^14^N-derivatized FA standards, including saturated FA and unsaturated FAs, at 4°C were screened at 0, 4, 8, 16, 24, 32, and 48 h after derivatization.

To evaluate matrix effects (ME), the DMAQ-^13^C/^15^N-FAs solution was spiked into ACN and DMAQ-^12^C/^14^N-derivatized meat extract, respectively. The final concentration of DMAQ-^13^C/^15^N-FAs in resulting solution was 100 ng/mL. The MEs were obtained by comparing the peak areas of derivatized FAs spiked in meat extracts with the ones in ACN. For the recovery study, meat sample was extracted using ACN containing 5 ng/mL of DMAQ-^13^C/^15^N-FAs standards. The resultant mixture was derivatized by DMAQ-^12^C/^14^N derivatization reagents. Recovery was demonstrated as (tested concentrations/theoretical concentrations) × 100%. Three replicates for each measurement were carried out.

### Data processing and statistical analysis

The peak integration was conducted using Multiquant 3.0 software (SCIEX, Framingham, MA, United States). In this study, the peak areas and FA concentrations were represented as mean ± standard deviation (SD). The differences in FA concentrations between meat under different harvest processes were estimated by students’ *t*-test using IBM SPSS 18.0 (SPSS Inc., Chicago, IL, United States). Statistical significances were considered if *p*-value was lower than 0.05. The graphs were produced using GraphPad Prism 8.0.2 and R 4.1 with Factoextra and Pheatmap packages.

## Results and discussion

**FIGURE 1:**
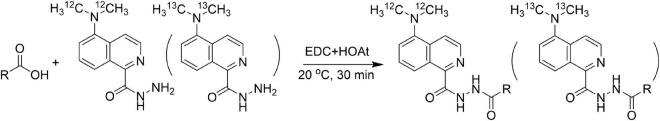
The reaction mechanism of carboxylic group with 5-(dimethylamino)-1-carbohydrazide-isoquinoline (DMAQ) under mild conditions.

Using LC-MS analysis of FAs is a challenge due to poor ionization efficiency brought by carboxyl species (14). Poor detection sensitivity made FAs analysis more critical especially for trace-amount FAs such as odd-chain and cyclic FAs in biological samples. In addition, the irregular dehydration of carboxy group [M–H_2_O–H]^–^ in ESI negative mode make the quantitative results unreliable. Also, the weakness in differentiating isomer FAs in RPLC should be considered. An alterable way to solve the above-mentioned problems was derivatization (4, 15). In this study, we developed a chemical isotopic derivatization method to accurately and sensitively quantify FAs based on an pair of DMAQ-^12^C/^14^N and DMAQ-^13^C/^15^N (Supplementary Figure 1), which had been reported in our previous work (13). In this derivatization reaction (Figure 1), carboxylic group was activated by carbodiimide, which was subsequently attacked by nucleophilic amine group from DMAQ to form amide bond.

### Optimization of derivatization reaction

As mentioned above, the derivatization reaction is based on carbodiimide-mediated coupling reaction. To reach the maximum derivatization efficiency, a mixed FAs standards solution containing seven FAs, schematically shown in Supplementary Figure 2, was employed. The coupling reaction was optimized regarding coupling-reagent types and their contents, additive types and their levels, DMAQ contents, time, and temperature by single-factor experiments. The peak areas of DMAQ-^12^C/^14^N-derivatized FAs were employed to evaluate the derivatization efficiency of different reaction conditions (Figure 1).

**FIGURE 2:**
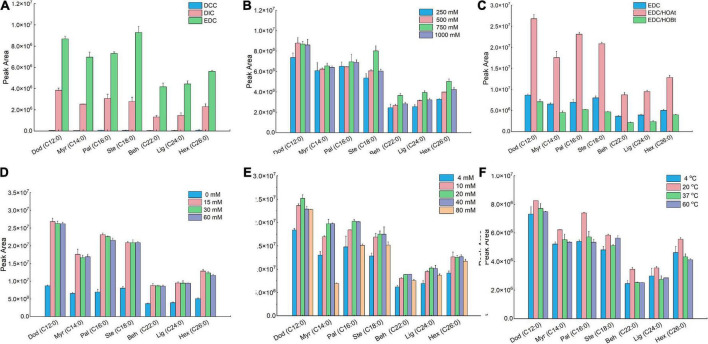
Comparison of derivatization efficiency of fatty acids (FAs) standard mixture under different reaction conditions. Effect of **(A)** coupling-reagent types; **(B)** ethylcarbodiimide hydrochloride (EDC) concentrations; **(C)** additive types; **(D)** HOAt concentrations; **(E)** 5-(dimethylamino)-1-carbohydrazide-isoquinoline (DMAQ) concentrations, and **(F)** temperature. Data were represented as the mean ± standard deviation (*n* = 3).

**FIGURE 3:**
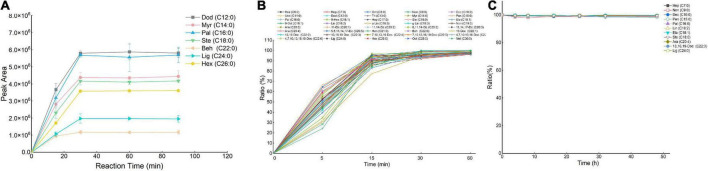
Effect of conversion efficiency of fatty acids (FAs) standards **(A)** and FAs in meat **(B)**. The stability study **(C)** of 5-(dimethylamino)-1-carbohydrazide-isoquinoline (DMAQ)-derivatized FAs at 4°C. Data were represented as the mean ± standard deviation (*n* = 3).

We first examined the effect of the coupling-reagent types on the derivatization efficiency (Figure 2A). In this study, three types of coupling reagents were tested including EDC, DCC, and DIC. For all seven FAs, the reaction using EDC behaved the best compared with that using DCC or DIC. Within the tested concentration range (Figure 2B), 750 mM of EDC provided better derivatization efficiency and therefore was selected as the optimal condition. To enhance the derivatization efficiency and reduce the side reactions, two additives including HOAt and HOBt were validated. We found that for all seven FAs, the derivatization solutions containing EDC/HOAt provided the highest derivatization efficiency (Figure 2C). We also examined the influence of HOAt concentration on the derivatization efficiency (Figure 2D). The results indicated that the addition of HOAt significantly enhanced the derivatization efficiency compared with that using EDC alone. However, there was no significant increase in derivatization efficiency while increasing HOAt concentration from 15 to 60 mM. Collectively, 15 mM of HOAt was chosen for subsequential analysis. For DMAQ concentration screening (Figure 2E), we found that the optimal DMAQ concentration was 20 mM. Other two parameters influencing the derivation efficiency were reaction temperature (Figure 2F) and time (Figure 3A). The results suggested that the optimized reaction conditions were carried out at 20°C for 30 min using the derivatization solution containing 750 mM EDC, 15 mM of HOAt, and 20 mM of DMAQ.

Considering the influence of other biomolecules on the derivatization reaction, a meat sample was employed as a complicated biological model to further determine whether the optimized derivatization conditions were applicable to a real biological sample. We validated the influence of EDC (Supplementary Figure 3A), HOAt (Supplementary Figure 3B), and DMAQ (Supplementary Figure 3C) concentrations on conversion efficiency. The optimal reaction was carried out using the derivatization solutions containing 750 mM of EDC, 15 mM of HOAt, and 20 mM of DMAQ, which were well agreed with that of FA standards labeling reaction. For the reaction temperature (Supplementary Figure 3D) and time (Figure 3B) screening, incubating at 20°C for 30 min can achieve the maximum conversion efficiency. Based on this, the optimal reaction conditions for FAs standards can be applicable to meat sample. However, for other types of biological sample such as cells, it is best to finetune the reagent concentration and reaction time to achieve the maximum conversation efficiency.

### General multiple reaction monitoring parameters

**FIGURE 4:**
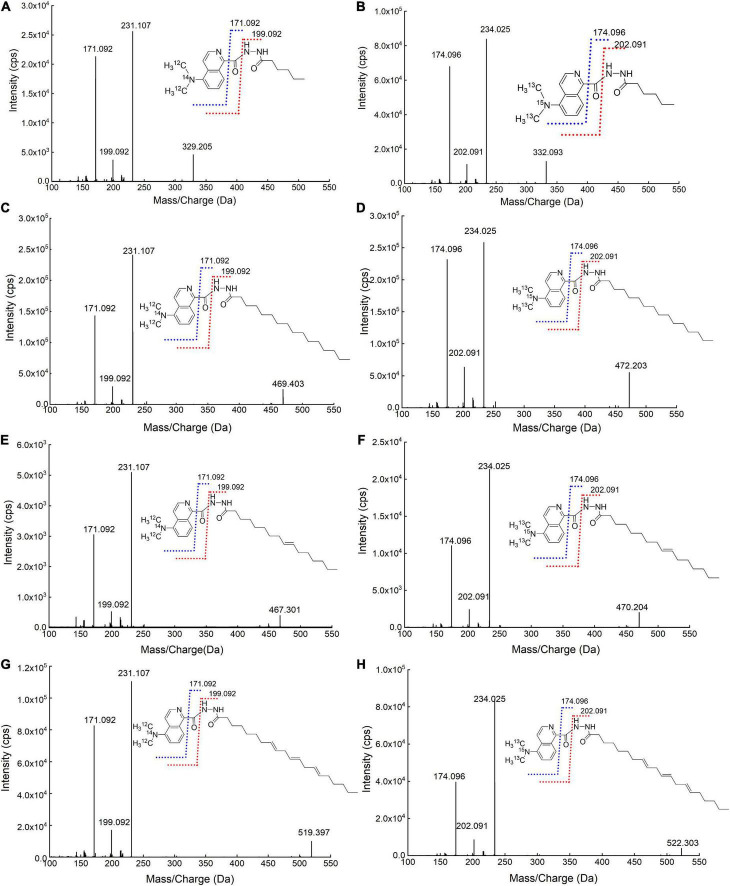
Mass spectrometry (MS)/MS spectra of **(A)** DMAQ-^12^C/^14^N-derivatized hex (C6:0); **(B)** DMAQ-^13^C/^15^N-derivatized hex (C6:0); **(C)** DMAQ-^12^C/^14^N-derivatized pal (C16:0); **(D)** DMAQ-^13^C/^15^N-derivatized pal (C16:0); **(E)** DMAQ-^12^C/^14^N-derivatized 9-Hex (C16:1); **(F)** DMAQ-^13^C/^15^N-derivatized 9-Hex (C16:1); **(G)** DMAQ-^12^C/^14^N-derivatized-8, 11, 14-Eic (C20:3), and **(H)** DMAQ-^13^C/^15^N-derivatized-8, 11, 14-Eic (C20:3).

To study the fragmentation patterns of DMAQ-derivatized FAs and identify the possible product ions for MRM monitoring, the MS/MS products of light-SFAs (hexanoic acid and palmitic acid) and UFAs (cis-9-hexadecenoic acid and cis-8, 11, 14-eicosatrienoic acid) were screened using high-resolution instrumentation (TOF-MS). As shown in Figure 4, the product ions at *m/z* 171.0922 and 199.0922 were observed for all DMAQ-^12^C/^14^N-FAs. The fragments were produced by breaking carbon-carbon bond adjacent to the isoquinoline skeleton and carbon–nitrogen bond in hydrazide group during collision-induced dissociation (CID), respectively. Similar phenomena were also observed for analyzing DMAQ-^13^C/^15^N-FAs by MS/MS. The product ions at *m/z* 174.0960 and 202.0909 related to heavy-FAs were observed in MS/MS spectra. Based on these, MRM conditions were individually optimized for all DMAQ-derivatized FAs. The optimal CE and DP of DMAQ-^12^C/^14^N- and DMAQ-^13^C/^15^N-FA standards (Supplementary Table 2) were constant, suggesting that the fragment patterns would not be affected by carbon chain length and the unsaturation degree in the structure. Hence, the optimized CE and DP can be applied to analyze other FAs. Compared with DMAQ-^12^C/^14^N-FAs, the *m/z* of precursor and product ions were increased by 3 Da for DMAQ-^13^C/^15^N-FAs since ^12^C and ^14^N from dimethylamine group were replaced by their isotopic analogs (^13^C and ^15^N). This difference mass can diminish the overlap of isotope peaks between DMAQ-^12^C/^14^N-FAs and DMAQ-^13^C/^15^N-FAs (13). For example, the theoretical isotope distributions of DMAQ-^12^C/^14^N-Hex (C6:0) were 328.19 (100%), 329.19 (21.8%), 330.20 (2.7%), and 331.20 (0.2%) which is only 0.2% overlapped with DMAQ-^13^C/^15^N-Hex (C6:0) first isotopic peak (331.20). By using this method, the quantitation analysis of all FAs may be applied to the analytes containing carboxyl group even when the standards are not commercially available.

### Method validation

A series of experiments regarding linearity, sensitivity, accuracy and precision, recoveries, and stability were carried out to validate the established LC-MS method under optimized conditions. To evaluate the correlation of DMAQ-FAs concentrations and MS response signals, we analyzed ten-level DMAQ-^13^C/^15^N derivatized FA standards in neat organic solvent (Supplementary Table 3) and DMAQ-^12^C/^14^N derivatized meat extract (Table 1). As shown, all of DMAQ-FAs demonstrated excellent calibration linearities over a wide dynamic range with correlation determinations (R^2^) greater than 0.99. The linear ranges of quantitation in both organic solvent and meat extract were determined to be 0.5–1,000 μg/L. The LOD and LOQ of DMAQ-FAs in meat extract were in the range of 5–75 and 10–100 ng/L, respectively, while that in ACN were well below 50 and 100 ng/L, respectively. These values are lower than those reported in previous works in which FAs were tested using GC-FID (Supplementary Table 2) (16). The accuracy and precision of the proposed method was accessed using intra- and inter-day assay, summarized in Table 1. For inter- and intra-day comparison experiments, the RSDs values of FAs were well below 3.74 and 5.72%, respectively. Recovery results for DMAQ-FAs were ranging from 80.20 to 99.50%. The product stability was tested using DMAQ-^12^C/^14^N-derivatized FAs standards (Figure 3C). Considering the structural differences, eight SFAs (e.g., C7:0 and C24:0) and four UFAs (C18:1 and C22:3) were chosen. There were no significant changes in peak areas of DMAQ-^12^C/^14^N -derivatized FAs observed, suggesting that DMAQ-^12^C/^14^N-derivatized FAs was stable for 48 h at 4°C and met the requirements of long-term instrumental analysis. Collectively, the established DMAQ derivatization method can be applied to the quantitative analysis of FAs with good linearity, sensitivity, accuracy, precision, and recovery. Good stability allowed the DMAQ-derivatized FAs for long-term instrument analysis.

**TABLE 1:** The linear range, calibration curve, LOD, LOQ, inter- and intra-day assays, matrix effect (ME) and recovery of 5-(dimethyamino)-1-carbohydrazide isoquinoline (DMAQ)-FAs in meat extract.

Analytes	Meat extract					RSD (%)		ME (%)	Recovery (%)
	Linear range (μg/L)	Regression equation y=a*x* + b	R^2^	LOD (ng/L)	LOQ (ng/L)	Inter-day (*n* = 3)	Intra-day (*n* = 9)		
Hexanoic acid	0.5–1,000	*y*=6.12*e*^4^*x* + 1.14*e*^4^	0.9997	10	50	2.9	4.56	45	92.2
Heptanoic acid	0.5–1,000	*y*=1.48*e*^5^*x* + 3.31*e*^5^	0.9997	15	30	2.25	2.27	55	99.5
Octanoic acid	0.5–1,000	*y*=9.26*e*^4^*x* + 1.15*e*^4^	0.9997	15	25	2.26	3.25	51	95.7
Nonanoic acid	0.5–1,000	*y*=2.31*e*^5^*x* + 3.16*e*^5^	0.9993	5	10	1.4	1.81	47	98
Decanoic acid	0.5–1,000	*y*=1.25*e*^5^*x* + 1.31*e*^5^	0.999	10	30	2.39	4.43	47	95.2
Undecanoic acid	0.5–1,000	*y*=8.82*e*^5^*x* + 1.32*e*^4^	0.9966	10	50	1.33	1.41	63	95.9
Dodecanoic acid	0.5–1,000	*y*=1.03*e*^5^*x* + 1.84*e*^4^	0.9955	5	50	2.72	4.76	74	87.2
Tridecanoic acid	0.5–1,000	*y*=8.17*e*^4^*x* + 2.93*e*^4^	0.996	15	40	1.8	3.14	82	92.2
Myristic acid	0.5–1,000	*y*=1.17*e*^5^*x* + 1.27*e*^5^	0.9979	10	40	1.07	4.51	64	98
Pentadecanoic acid	0.5–1,000	*y*=2.82*e*^4^*x* + 3.42*e*^5^	0.9984	50	100	3.17	3.49	56	94.7
Palmitic acid	0.5–1,000	*y*=2.67*e*^4^*x* + 2.77*e*^5^	0.9987	5	25	2.75	3.41	64	95.2
cis-9-Hexadecenoic acid	0.5–1,000	*y*=7.04*e*^4^*x* + 8.35*e*^3^	0.9983	15	40	3.74	4.68	79	96.5
Heptadecanoic acid	0.5–1,000	*y*=1.05*e*^4^*x* + 1.59*e*^5^	0.9994	15	50	2.37	3.69	70	90
Stearic acid	0.5–1,000	*y*=1.21*e*^4^*x* + 1.96*e*^5^	0.9986	25	50	2.18	4.1	58	96.4
Elaidic acid	0.5–1,000	*y*=1.25*e*^4^*x* + 2.18*e*^5^	0.9994	25	50	3.23	3.39	68	91
cis-9-Octadecenoic acid	0.5–1,000	*y*=5.41*e*^4^*x*−9.54*e*^3^	0.9992	30	75	3.53	5.72	68	84.1
Linoleic acid	0.5–1,000	*y*=5.40*e*^4^*x* + 4.24*e*^4^	0.9983	5	25	2.7	1.88	75	94.3
a−Linoleic acid	0.5–1,000	*y*=9.43*e*^3^*x* + 2.52*e*^4^	0.9978	25	70	1.39	4.81	63	85.3
g−Linoleic acid	0.5–1,000	*y*=6.09*e*^4^*x* + 5.61*e*^5^	0.9984	5	25	3.04	4.19	59	87.7
Nonadecanoic acid	0.5–1,000	*y*=1.12*e*^4^*x* + 1.20*e*^5^	0.9987	15	50	1.34	2.05	75	94.2
Arachidic acid	0.5–1,000	*y*=4.34*e*^4^*x* + 6.84*e*^4^	0.9994	5	35	1.96	3.49	77	92.9
cis-11-Eicosenoic acid	0.5–1,000	*y*=8.90*e*^4^*x* + 1.22*e*^5^	0.9991	30	100	2.96	4.68	69	84.2
cis-11,14-Eicosadienoic acid	0.5–1,000	*y*=7.27*e*^4^*x* + 1.18*e*^5^	0.9994	15	50	0.73	4.83	69	81.5
cis-8,11,14-Eicosatrienoic acid	0.5–1,000	*y*=1.41*e*^4^*x* + 4.41*e*^5^	0.9978	30	100	2.98	2.63	60	98.8
11-cis,14-cis,17-cis-Eicosatrienoic acid	0.5–1,000	*y*=5.12*e*^3^*x* + 9.69*e*^3^	0.9995	25	75	2.8	2.9	38	80.2
Arachidonic acid	0.5–1,000	*y*=8.44*e*^3^*x* + 7.39*e*^3^	0.9986	30	100	2.67	5.13	70	94.1
cis-5,8,11,14,17- Eicosatrienoic acid	0.5–1,000	*y*=2.54*e*^4^*x* + 1.96*e*^4^	0.9978	75	100	3.35	2.44	85	97.2
Heneicosanoic acid	0.5–1,000	*y*=1.03*e*^4^*x* + 7.80*e*^4^	0.9975	10	50	2.17	4.24	95	93.6
Behenic acid	0.5–1,000	*y*=1.03*e*^4^*x* + 7.80*e*^4^	0.9973	25	75	1.7	1.5	75	91.4
cis-13-Docosenoic acid	0.5–1,000	*y*=2.24*e*^3^*x* + 1.82*e*^5^	0.9982	10	50	3.07	2.44	76	94.3
cis-13,16-Docosadienoic acid	0.5–1,000	*y*=7.25*e*^4^*x* + 9.01*e*^4^	0.9989	20	50	1.59	1.78	88	93.1
cis-13,16,19-Docosatrienoic acid	0.5–1,000	*y*=7.87*e*^4^*x* + 1.24*e*^5^	0.9994	20	50	2.75	3.17	71	92.4
cis-7,10,13,16-Docosatetraenoic acid	0.5–1,000	*y*=7.16*e*^4^*x* + 1.11*e*^5^	0.9993	15	50	3.43	2.62	74	92.4
cis-7,10,13,16,19-Docosapentaenoic acid	0.5–1,000	*y*=5.68*e*^4^*x* + 8.19*e*^4^	0.9994	30	100	2.2	1.67	76	88.9
cis-4,7,10,13,16-Docosapentaenoic acid	0.5–1,000	*y*=2.20*e*^3^*x* + 3.42*e*^3^	0.9995	50	100	2.31	1.61	79	87.4
cis-4,7,10,13,16,19-Docosapentaenoic acid	0.5–1,000	*y*=2.14*e*^4^*x* + 2.62*e*^4^	0.999	25	75	2.5	2.74	65	96.6
Lignoceric acid	0.5–1,000	*y*=8.28*e*^4^*x* + 1.83*e*^5^	0.9991	15	100	1.87	4.02	64	91.1
Hexacosanoic acid	0.5–1,000	*y*=6.78*e*^4^*x* + 2.86*e*^5^	0.9971	15	100	3.63	3.54	62	92.4
Octacosanoic acid	0.5–1,000	*y*=7.88*e*^4^*x* + 1.83*e*^4^	0.9972	10	25	5.29	2.22	49	92.4
Melissic acid	0.5–1,000	*y*=1.47*e*^4^*x* + 5.06*e*^4^	0.9979	50	100	2.68	2.44	33	91.8

### Improvement on sensitivity and chromatography separation upon 5-(dimethyamino)-1-carbohydrazide isoquinoline derivatization

**FIGURE 5:**
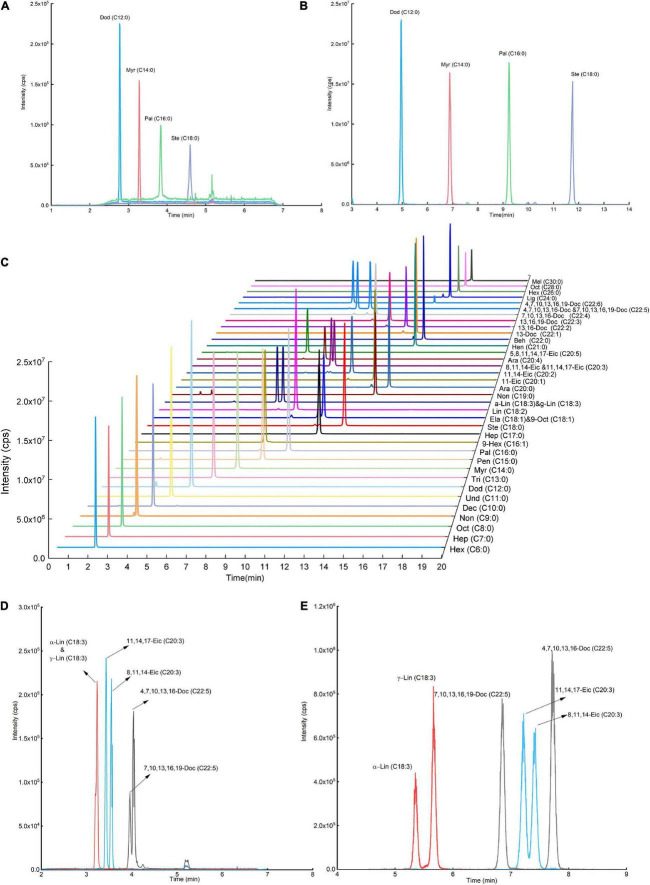
Sensitivity evaluation before **(A)** and after **(B)** fatty acid (FA) derivatization reaction. Liquid chromatograph (LC) chromatograms of **(C)** 5-(dimethyamino)-1-carbohydrazide isoquinoline (DMAQ)-derivatized FA standards with different carbon-chain length. The relationship LC chromatograms of isomer FA standards **(D)** before and **(E)** after derivatization.

After derivatization, we compared the MS signal response of DMAQ derivatized and underivatized FAs. The significant enhancements in signal responses for DMAQ-derivatized FAs were demonstrated (Figures 5A,B), benefiting from heteroatom (nitrogen atom) in tertiary amine and dimethylamine groups. Additionally, DMAQ derivates can be detected under positive ESI mode as four easy-ionizable nitrogen atoms in DMAQ which remarkably improved ionization efficiency. In complex biological matrix, the LODs for forty FAs, shown in Table 1, was well below 75 ng/L. As the high sensitivity, the low contents of free and esterified odd-chain fatty acids were successful quantified in meat (Supplementary Tables 4, 5).

The separation ability of reversed-phase LC (RPLC) is strongly correlated with hydrophobic properties of FAs. Their hydrophobic properties are determined by the number of aliphatic carbons, unsaturation degrees and the location of double bond. FAs have a wide range of hydrophobicity due to the aliphatic carbon from C6 to C30. To be able to separate the FAs in RPLC, high slope of organic phase ratio or prolonging separation time is employed. However, these two methods were not applicable to separate isomer FAs with similar hydrophobic properties, especially for double bond at different locations. The established DMAQ derivatization method successfully narrows range of hydrophobicity of FAs and benefits the separation performance of structural isomer FAs. As shown in Figures 5D,E, α-linolenic acid/γ-linolenic acid, cis-8,11,14-eicosatrienoic acid/11-cis, 14-cis, 17-cis-eicosatrienoic acid, and cis-4, 7, 10, 13, 16-/cis-7, 10, 13, 16, 19-docosapentaenoic acid, which have same molecular weight and only different in the double-bond position, were hard to separate in LC-MS before derivatization. After derivatization, the DMAQ-derivatized FAs were easily separated in C8 column. And forty DMAQ-derivatized FAs with different aliphatic carbons and unsaturated degree were completely separated in 20 min shown in Figure 5C. Quantification of VLFAs using GC was difficult due to the low volatility and limited separation efficiency on GC column. Upon DMAQ derivatization, all DMAQ-VLFAs (C26-C30 carbons) reached baseline separation with excellent linear relationship and improved sensitivity. Collectively, DMAQ derivatization can achieve good separation on column with improved sensitivity.

### Minimizing matrix effects using internal-standard method

**FIGURE 6:**
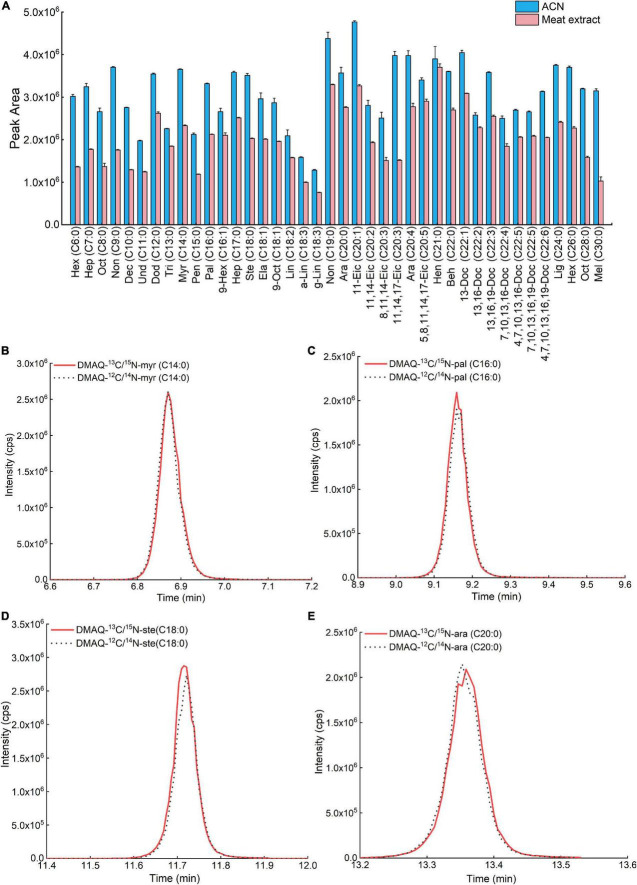
Comparison of **(A)** peak areas of fatty acids (FAs) in acetonitrile and biological matrix. TIC of **(B)** DMAQ-^12^C/^14^N- and DMAQ-^13^C/^15^N-derivatized-myr (C14:0), **(C)** DMAQ-^12^C/^14^N- and DMAQ-^13^C/^15^N-derivatized-pal (C16:0), **(D)** DMAQ-^12^C/^14^N- and DMAQ-^13^C/^15^N-derivatized ste(C18:0), and **(E)** DMAQ-^12^C/^14^N- and DMAQ-^13^C/^15^N-derivatized-ara (C20:0).

To quantify target compounds using MS, there is always a deviation between the tested value and true value, especially using calibration curve made directly in neat solvent. This was caused by other no-target compounds in coelution solution (17). Hence, we first evaluated the matrix effect of each DMAQ-^13^C/^15^N derivatives in meat extract, displayed in Figure 6A. The results demonstrated that the signal responses of most DMAQ-^13^C/^15^N-derivatized FAs were reduced by more than twenty percent, suggesting an inhibition effect (Table 1). To reduce matrix effects uncertainty, matrix-matched calibration solutions were frequently employed which extracted from blank sample including the same endogenous compounds without the analytes of interest (18). Since a proper blank matrix is hardly obtained, isotope-dilution mass spectrometry provides an alternative way (19). But the varieties of isotopic-coded compounds are limited availability, and their price are very expensive. Here, we reported an isotopic-coded DMAQ derivatization strategy that incorporated isotopic carbon and nitrogen atoms for the quantitative analysis of FAs. Figures 6B–E showed that DMAQ-^12^C/^14^N and DMAQ-^13^C/^15^N derivatized FAs eluted at the same time. Moreover, the tested peak area ratio of DMAQ-^12^C/^14^N and DMAQ-^13^C/^15^N derivatized FAs was close to the theoretical ratios with R^2^ of 0.99 from 100:1 to 1:100 (Supplementary Figure 4). The results suggests that the content of FAs in the biological samples can be measured based on the peak area ratios of DMAQ-^12^C/^14^N derivatized biological sample and DMAQ-^13^C/^15^N derivatized standards with known concentration. Based on these, the concentration of FAs in meat under different post-harvest process was accurately validated using internal-standard method.

### Fatty acid characterization of meat samples under different harvest treatment

**FIGURE 7:**
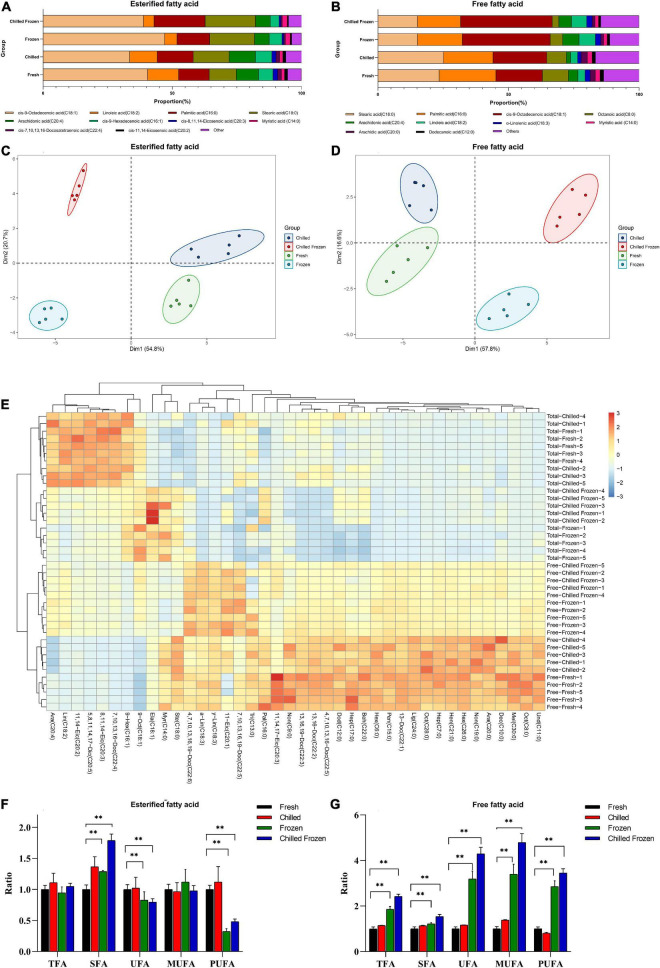
The ratio of **(A)** esterified FAs and **(B)** free FAs from meat under different postharvest processes; PCA-Biplot analysis of **(C)** free FAs and **(D)** esterified FA under various harvest processes; **(E)** the Heatmap of forty FAs from meat under various postharvest. The ratios of TFA, SFA, UFA, MUFA and PUFA in esterified FAs **(F)** and free FAs **(G)** from meat under different postharvest processes. Data were represented as the mean ± standard deviation (*n* = 5). **p < 0.01 was considered as significantly different.

To further validate method efficacy, we used this strategy to quantify the contents of free and esterified FAs in meat samples under different post-harvest processes, named Fresh, Chilled, Frozen, and Chilled Frozen (Supplementary Tables 4, 5). The representative chromatographs for both free and esterified FAs was shown in Supplementary Figure 5. In the following discussion, the tested esterified FAs include both free FAs and un-freed FAs which were linked with lipid. Since free FAs contents under 0.5% of un-freed FAs, its influence on the tendency of esterified FAs during different post-harvest can be neglected. As shown in Figure 7A, the top 10 of total FAs in meat were cis-9-octadecenoic acid, linoleic acid, palmitic acid, stearic acid, arachidonic acid, cis-9-hexadecenoic acid, cis-8,11,14-eicosenoic acid, cis-7,10,13,16-docosatetraenoic acid, myristic acid, and cis-11,14-eicosenoic acid. The proportion of those FAs were from 93.97 to 96.24% in four different post-harvested meats. The proportion of esterified FAs was well agreed with previous reports (16, 20). Comparing with esterified FAs, the proportion of free FAs was different. As shown in Figure 7B, the top 10 of free FAs in meat were stearic acid, palmitic acid, cis-9-octadecenoic acid, octanoic acid, arachidonic acid, linoleic acid, a-linolenic acid, arachidic acid, myristic acid, and dodecanoic acid. The proportion of those FAs were from 83.30 to 88.77% in four types of different post-harvested meats. Moreover, the proportions of esterified and free FAs in four post-harvest processes were significant changed. As shown in Figures 7C,D, unsupervised clustering analysis showed that the four groups were clearly distinguishable with good separation on the PCA scatter plot, suggesting that there were obvious changes in of both esterified and free FA during different post-harvest processes. The heatmap (Figure 7E) gave a clear pattern of the changed tendencies of FAs. As shown, the samples form Fresh and Chilled were clustered together, while the samples from Frozen and Chilled Frozen were clustered into same class. It meant that freezing process resulted in significant changes in both esterified and free FAs. In addition, the changed tendencies of some FAs were opposite comparing the esterified and free FAs during freezing process. To deeply investigate the change pattern, all the FAs were clarified and accumulated into five groups on the basis of the number of double bonds, including total FA (TFA), SFA, unsaturated fatty acid (UFA), MUFA, and PUFA. Comparing samples in Chilled, Frozen, and Chilled Frozen groups with that in Fresh group (Figures 7F,G), the esterified TFA and MUFA in four group were not significant changed (*p* > 0.05), while free TFA and MUFA in Frozen and Aged Frozen group were significant increased (*p* < 0.01). Both esterified and free SFA were increased after chilling or/and freezing process (*p* < 0.01). While the tendencies of free PUFA were completely opposite with esterified PUFA after freezing and aged freezing process, thereby the tendencies of UFA were also opposite. In meat, the presence of highly UFA and a large content of pro-oxidant molecules can lead to substantial enzymatic and non-enzymatic rancidity that have strongly influencing on the meat quality after chilled and frozen storage. The increase of free UFA, MUFAs, and PUFAs may be due to the complicated lipid hydrolysis caused by certain types of microbial or enzyme from neutral and polar lipids during the freeze-thaw process. The existence of oxidation indicator (radicals) can lead to the oxidative change (21). These changes have also been extensively explained by the enzyme oxidation or autoxidation of cis-9-octadecenoic acid (C18:1) and linoleic acid (C18:2) (22, 23). Esterified SFA contents were observed to be higher in both frozen and chilled frozen samples compared with their counterparts. Palmitic acid (C16:0) and stearic acid (C18:0) were the major contributor for Esterified SFA. Similar trends were observed in beef frozen for 12 months (24). Both Chilled and Chilled Frozen samples PUFAs were instead lower than their unfrozen one (p < 0.01). The changes of PUFAs can be attributed to lipid peroxide. The termination of the chain reaction leads to the fatty aldehyde, ketone, and other degradation components of oxidized FAs (25). Hence, the results suggest that the protocol outlined here could be successfully utilized to profile both FFAs and esterified FAs with diversity structure even at trace amount.

## Conclusion

In recent study, an isotopic-coded DMAQ-derivation coupled with LC-MS analysis was established for screening FAs with diversity structures. FAs derivatization using DMAQ was performed rapidly under mild condition. After derivatization reaction, both the cyclic skeletons and easy ionizable nitrogen atoms in DMAQ were confer to FAs, resulting in improved chromatograph performance. Meanwhile, the detection sensitivity of the FA derivatives was remarkably improved with the LOQ from 10 to 100 ng/L. Even using the biological matrix, the developed derivatization method has wide dynamic range of 0.5–1,000 μg/L with R^2^ greater than 0.99. The RSDs values for inter- and intra-day assays were well below 3.74 and 5.72%, respectively, indicating the developed method with good precision. Benefiting from the developed isotopic derivation method for FAs, matrix effects have been diminished using DMAQ-^13^C/^15^N-derivatized FAs as internal standards. This method has been successful verified using different post-harvest meats as biological models, and the results demonstrates that both the odd-chain FAs and VLFA have been detected, suggesting that the high sensitivity of the developed method. Comprehensive, isotopic-coded DMAQ-derivation coupled with LC-MS analysis is a powerful method for targeted analysis of FAs with high sensitivity and accuracy in biological systems especially for carboxyl-containing compounds at low abundance.

## Data availability statement

The original contributions presented in this study are included in the article/Supplementary material, further inquiries can be directed to the corresponding author.

## Ethics statement

The animal study was reviewed and approved by Science Research Department of Institute of Animal Sciences, Chinese Academy of Agricultural Sciences.

## Author contributions

XF: conceptualization, methodology, validation, and writing - original draft. JW and ZT: writing - review and editing. BC: investigation and validation. XH: sample collection and validation. JL and SF: validation. PL: sample collection, investigation, and validation. QM: conceptualization, methodology, writing - original draft, writing - review and editing, and funding acquisition. All authors contributed to the article and approved the submitted version.
