# Supplementary material for: A strategy for accurately and sensitively quantifying free and esterified fatty acids using liquid chromatography mass spectrometry
Source: Front Nutr. 2022 Aug 3;9:977076. doi: 10.3389/fnut.2022.977076 (PMC9381817; doi:10.3389/fnut.2022.977076)

**Supplemental Information**

**A Strategy for Accurately and Sensitively Quantifying** **Free and Esterified Fatty Acids** **using Liquid Chromatography Mass Spectrometry**

Xiaohui Feng^a, †^, Juan Wang^b, †^, ,Zhonghai Tang^c^, Bingyao Chen^a,c^, Xinhua Hou^a^, Jing Li^a^, Shengnan Feng^a^, Peng Li^d^, Qingshi Meng^a,^ *

^a^ A State Key Laboratory of Animal Nutrition, Institute of Animal Sciences, Chinese Academy of Agricultural Sciences, Beijing, 100193, China

^b^ Biotechnology Research Institute, Chinese Academy of Agricultural Sciences, Beijing, 100081, China

^c^ College of Food Science and Technology, Hunan Agricultural University, Changsha, 410128, China

^d^ China Animal Disease Control Center, Beijing, 100125, China

^†^ These two authors contributed equally to this work.

***Please address all correspondence to:**

Qingshi Meng

Email address: mengqingshi@caas.cn (Q. Meng) Tel: +86 10 62815859

Postal address: Institute of Animal Sciences, No.2 Yuamingyuan West Road, Beijing 100193, China

**Table of Contents**

1. **Supplementary Figures**

Fig. S1 Chemical Structures for (A) DMAQ-^12^C/^14^N and (B) DMAQ-^13^C/^15^N…….……..……………3

Fig. S2 Structures of seven FA standards used to form a mixed standards solution for derivatization reaction optimization………………………………………………………………………… ………...4

Fig. S3 Comparison of derivatization efficiency of meat extract under different reaction conditions. Effect of (A) EDC concentrations; (B) HOAt concentrations; (C) DMAQ concentrations; (D) temperature. Data were represented as the mean ± standard deviation (n=3)…………………..……..5

Fig. S4 The linear regression of DMAQ-^12^C/^14^N- and DMAQ-^13^C/^15^N-derivatizaed forty FA standards…………..…………………………………………………………………….………………6

Fig. S5 LC chromatograms of (A) free FAs and (B) esterified FAs extracted from meat were derivatized by DMAQ-^12^C/^14^N-FAs and mixed with mixed with DMAQ-^13^C/^15^N-coded IS.

1. **Supplementary Figures**

Fig. S1 Chemical Structure for (A) DMAQ-^12^C/^14^N and (B) DMAQ-^13^C/^15^N





Fig. S2 Structures of seven FA standards used to form a mixed stock solution for derivatization reaction optimization

**

**

Fig. S3 Comparison of derivatization efficiency of meat extract under different reaction conditions. Effect of (A) EDC concentrations; (B) HOAt concentrations; (C) DMAQ concentrations and (D) temperature. Data were represented as the mean ± SD (n=3).

**
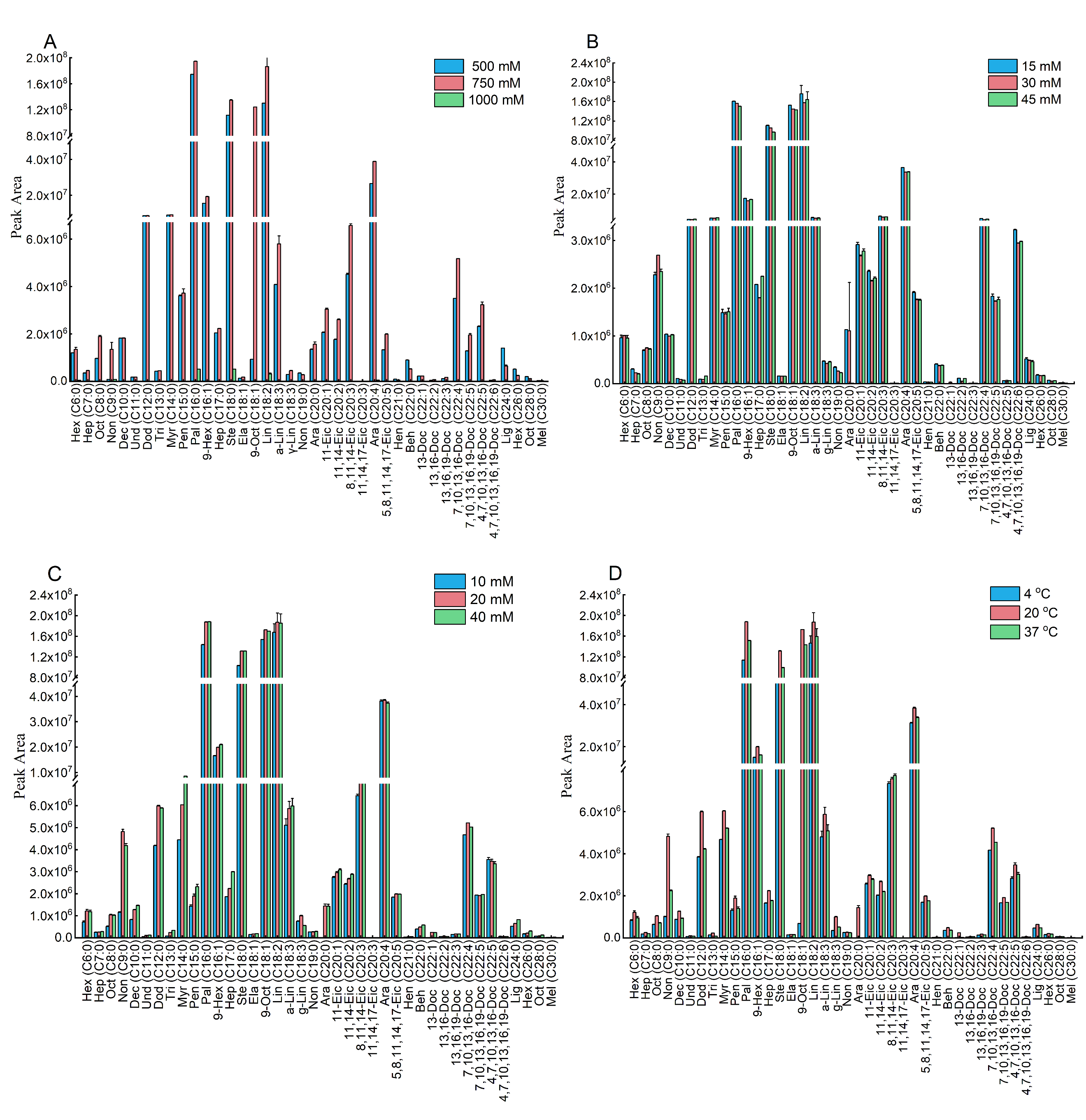
**

Fig. S4 The linear regression of DMAQ-^12^C/^14^N- and DMAQ-^13^C/^15^N-derivatized forty FA standards

**
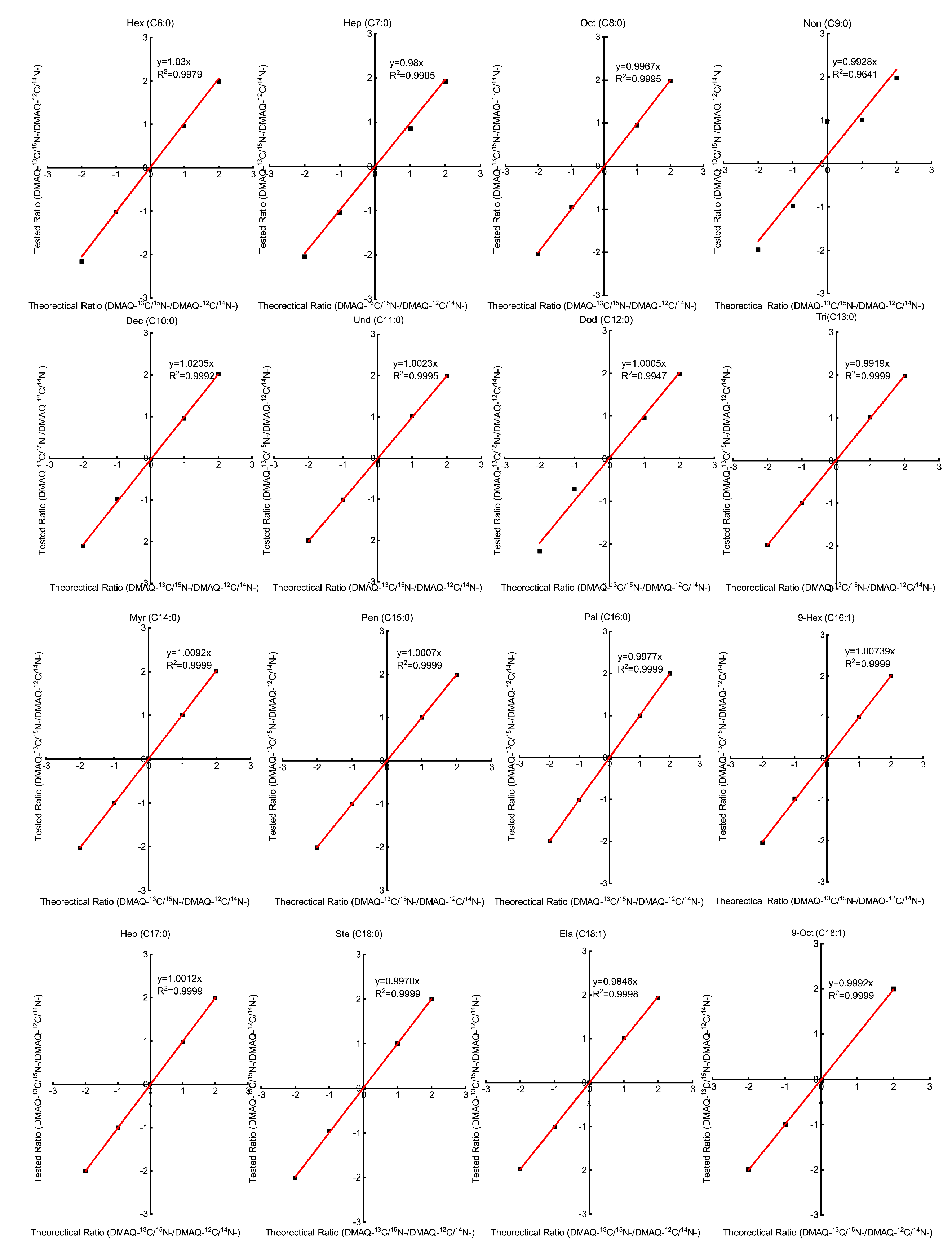
**


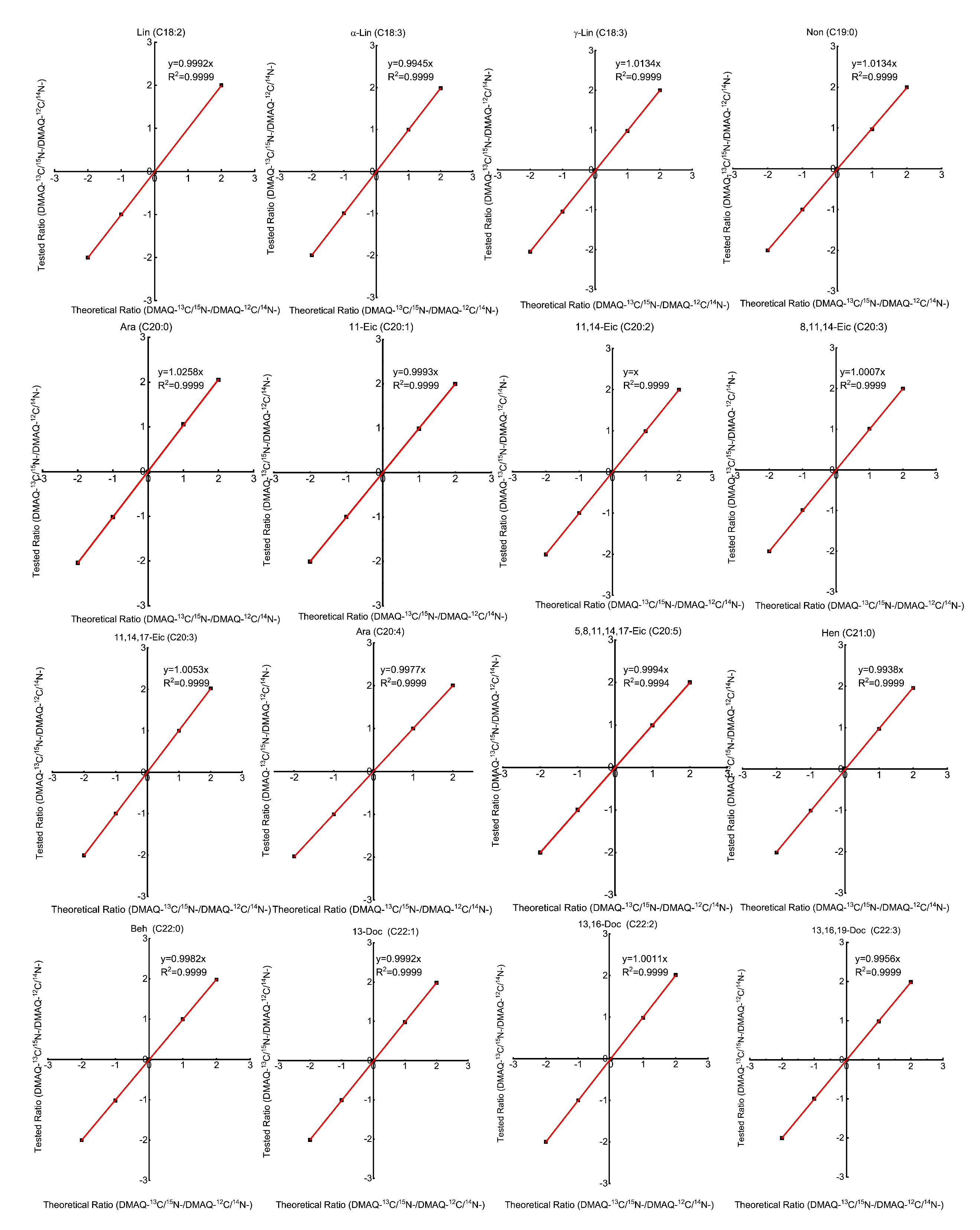


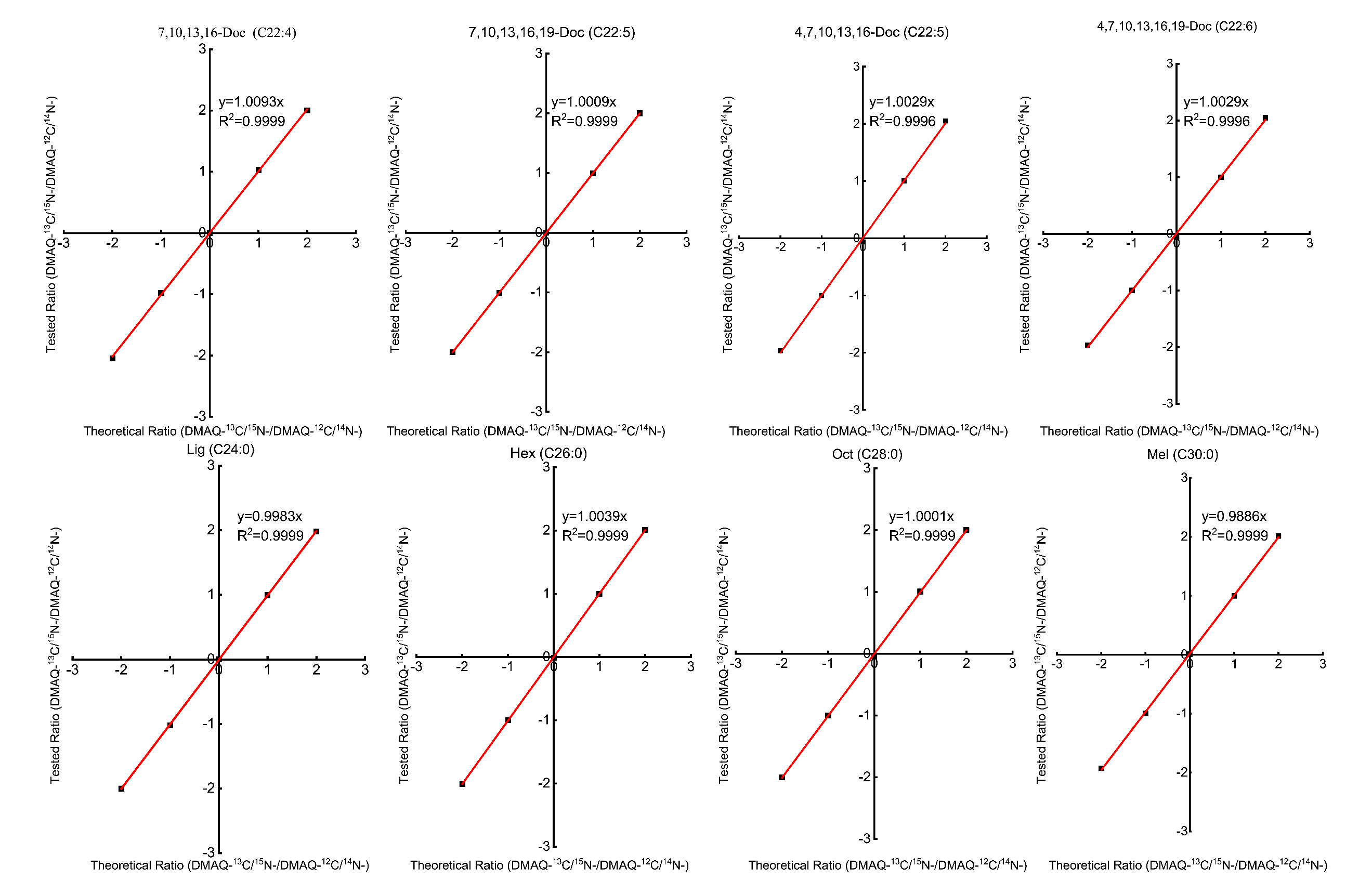


Fig. S5 LC chromatograms of (A) free FAs and (B) esterified FAs extracted from meat were derivatized by DMAQ-^12^C/^14^N-FAs and mixed with mixed with DMAQ-^13^C/^15^N-coded IS.


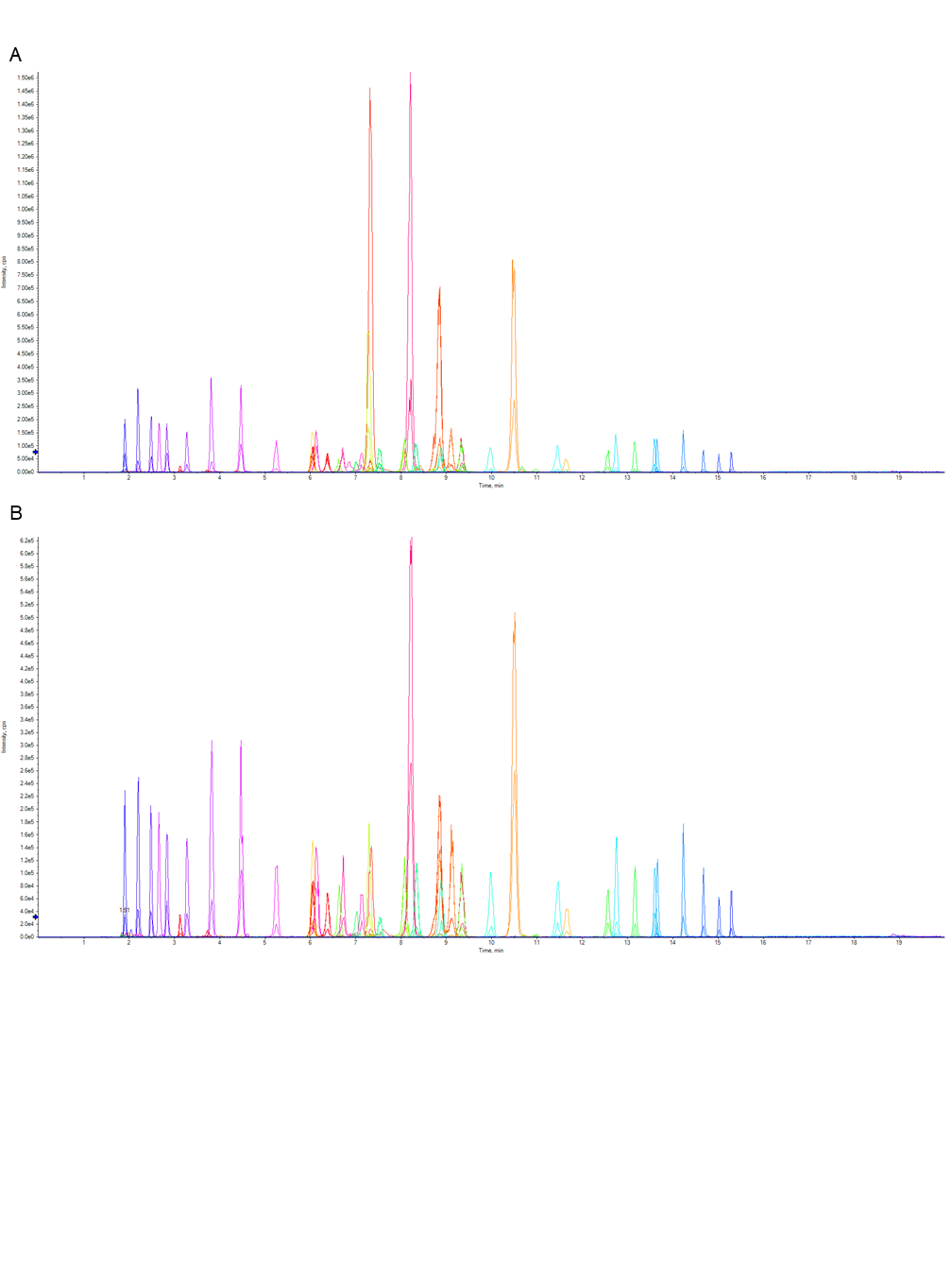

Supplement: Supplementary file 1 [file Data_Sheet_1.docx]
